# A Novel Zinc-Containing Palatal Stent and Topical Oxygen Therapy for Wound Protection and Healing Following Mucoperiosteal Flap Surgery in the Hard Palate: A Case Report

**DOI:** 10.7759/cureus.64095

**Published:** 2024-07-08

**Authors:** Minas Leventis, Kenneth Van Stralen

**Affiliations:** 1 Oral Surgery, Dental School, National and Kapodistrian University of Athens, Athens, GRC; 2 Dentistry, Marquette University, Milwaukee, USA

**Keywords:** palatal stent, zinc-embedded polymer, palatal surgery, nasopalatine duct cyst, alloplastic bone graft, oxygen therapy, wound healing

## Abstract

In oral surgery, common surgical procedures such as the removal of impacted teeth, the treatment of intraosseous cysts and tumors, and endodontic surgery often require access through a palatal approach. Full-thickness flap surgery in the hard palate region can result in significant post-operative pain, swelling, and hematoma, adversely affecting the patient's function and well-being for several days. Moreover, post-operative infection can delay or compromise healing. Post-surgical traditional palatal stents have been shown to effectively reduce discomfort by minimizing swelling and pain during the early healing phases.

Recent advances in materials with the incorporation of bioactive agents have led to the fabrication of a new generation of wound dressings that provide improved conditions for effective wound protection and healing. This case report illustrates the use of a novel, zinc-embedded, thermoplastic surgical polymer for the chairside fabrication of post-operative palatal stents. A 33-year-old female patient, who underwent mucoperiosteal flap surgery for the management of a nasopalatine duct cyst, was provided immediately post-surgery with a customized zinc-containing palatal stent. The bone defect was grafted using a fully resorbable synthetic bone substitute, and an oxygen and lactoferrin-releasing oral gel was provided post-operatively as an adjunct therapy. The innovative stent helped the patient maintain low levels of pain and minimal swelling during the initial post-operative period, resulting in uneventful healing, as documented during the one-week follow-up appointment. Further reviews at four weeks and six months post-surgery revealed successful healing and sensory recovery in the anterior palatal region.

As emphasized in this report, the chairside fabrication of zinc-containing palatal stents for post-operative wound protection seems to constitute a valid, simple, time-saving, and cost-effective clinical solution. Moreover, the incorporation of zinc nanoparticles into the stent is of great clinical importance, potentially offering significant benefits in post-operative bacterial control and enhancement of the early-phase palatal soft-tissue healing.

## Introduction

A palatal stent is a device used to support and protect the palate, particularly after surgery or injury, made from biocompatible materials. Key characteristics of a post-operative palatal stent include 1) adequate mechanical retention, 2) effective protection of the surgical area, 3) prevention of hematoma and dead space formation, 4) durability to withstand daily activities, 5) ease of fabrication and repair for cost-efficiency, and 6) user-friendly design to optimize its utility as a surgical aid. Palatal stents are essential tools in post-operative care in dentistry as they can reduce discomfort by decreasing pain and alleviating swelling after surgery in the hard palate region [1-3]. Modified Essix and Hawley retainers, resin, and silicone palatal stents may protect the site post-surgery, while the gentle direct pressure exerted by a rigid splint aids in homeostasis and clot formation. Continued splint wear during the initial healing period stabilizes the coagulum and protects the site from the environment, minimizing post-operative patient discomfort. In a clinical study, Jang et al. showed that the use of palatal stents following mesiodens removal improved safety, enabled rapid healing by protecting the surgical site from irritation, and reduced pain and swelling by compressing the edema [3]. In a study by Basma et al., collagen plugs with sutures, collagen plugs with cyanoacrylate, platelet-rich fibrin with sutures, and palatal stents were compared to determine which of the four best minimized post-operative patient discomfort. The authors concluded that palatal stents exhibited the lowest overall pain scores over the critical two-week post-operative healing period [2,3].

Incorporating bioactive agents, such as zinc, into the palatal stent's material, may significantly improve the splint's properties. Zinc is a biodegradable metal, increasingly utilized in medicine due to its ability to accelerate wound healing and effectively prevent and treat bacterial infections [4]. Zinc, being an essential trace element and micronutrient, plays pivotal roles in human physiology, including growth, development, bone metabolism, immune function, and wound healing, and its deficiency can delay and compromise healing. Several studies have demonstrated that zinc exerts significant effects on various cells throughout the wound repair process and regulates every phase of wound repair, from membrane repair and oxidative stress management to coagulation, inflammation, immune defense, tissue re-epithelialization, angiogenesis, and fibrosis/scar formation. It enhances platelet activity and aggregation following injury, promoting hemostasis through coagulation and clot formation. Zinc concentration is critical in promoting all aspects of the inflammatory phase. It supports neoangiogenesis by stimulating endothelial cell migration and proliferation, improving the establishment of new blood vessels to supply oxygen and nutrients essential for cellular growth within the wound bed. Additionally, zinc plays a crucial role in wound closure as it facilitates the formation of granulation tissue and the proliferation of fibroblasts, increasing collagen synthesis. It modulates integrins expressed by the basal layer of keratinocytes, enhances their cellular mobility, and stimulates their proliferation, thus supporting re-epithelialization and speeding up the proliferation phase of healing of cutaneous and oral wounds [5].

Furthermore, zinc exhibits antibacterial properties that effectively inhibit the formation of gingivitis, plaque, and calculus, thus minimizing the risk of post-operative wound infection. Zinc ions specifically bind to thiol groups present in bacterial respiratory enzymes, thereby compromising their activity. Additionally, zinc has been shown to inhibit active transport processes across bacterial plasma membranes and interfere with amino acid metabolism [6-8].

The aim of this case report is to present the use of a novel chairside-made palatal stent composed of thermoplastic zinc-infused polymer granules, in synergy with topical oxygen therapy, to protect the wound and promote the healing after mucoperiosteal flap surgery in the hard palate region.

## Case presentation

**Figure 1:**
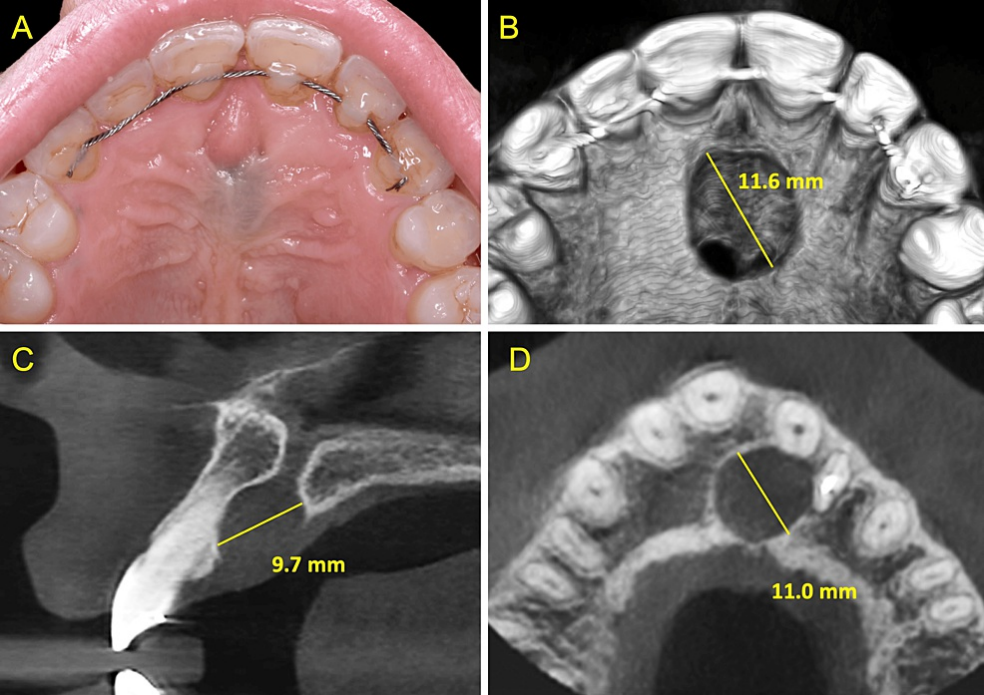
Initial clinical (A) and radiological (B-D) views

A 33-year-old female patient presented with an asymptomatic swelling in the central region of the palate. The patient's general dentist initially identified the lesion one year ago. The medical history was non-contributory. According to the dental history, orthodontic treatment was undertaken in the past, at the age of eight, involving palatal expansion. Four months prior to presentation, the patient experienced persistent spontaneous localized pain when chewing and sensitivity in the upper left incisor (#22), which endodontic treatment did not resolve. Extraoral clinical examination showed no abnormalities. Intraoral clinical evaluation revealed a soft, bluish, fluctuant swelling in the midline of the hard palate, without discharge, fistula, or other signs of acute or chronic infection. The maxillary teeth exhibited no mobility, deep pocketing, or periodontal inflammation. Pulp vitality tests showed that all upper anterior teeth were vital, except for endodontically treated #22 which was sensitive to percussion. There was no bleeding on probing, or periodontal pathology associated with #22, nor any other signs indicative of acute infection or tooth fracture. A fixed wire retainer was bonded on the upper anterior teeth. Cone beam computed tomography (CBCT) revealed a well-demarcated, round, symmetric radiolucency, measuring 11.6 mm in diameter. This radiolucency demonstrated continuity with the incisive canal, as well as with a localized periapical radiolucency associated with the symptomatic, root canal-treated #22 (Figure 1).

**Figure 2:**
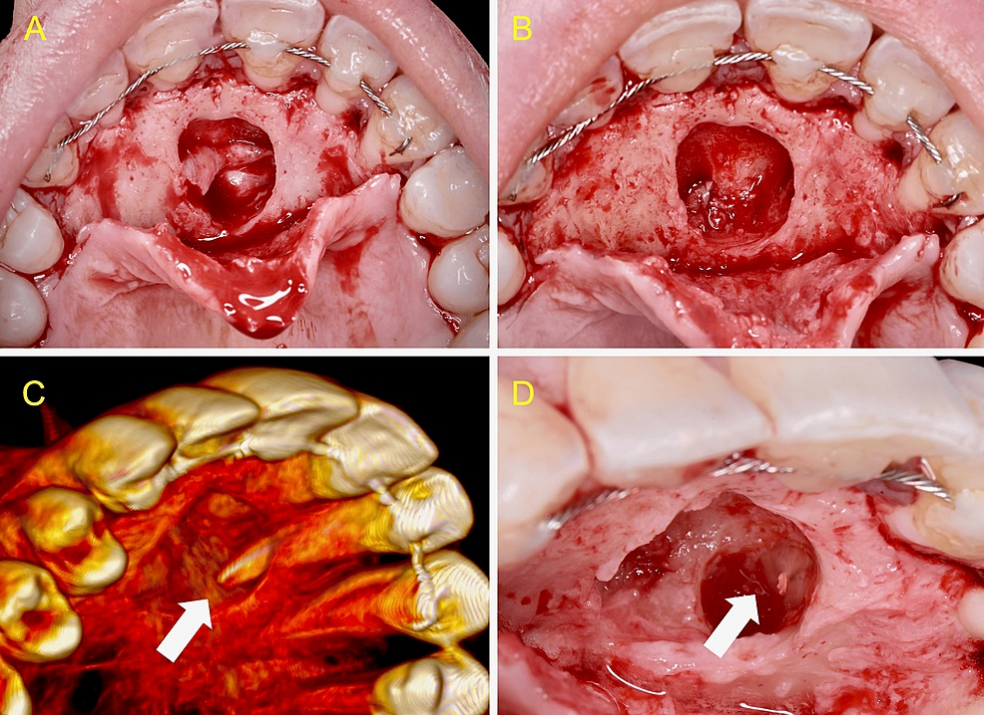
Surgical debridement of the cystic lesion A: A full-thickness palatal flap was raised B: The cystic lesion was enucleated C: Radiological view of the associated periapical lesion (arrow) D: The apex of #22 was resected, followed by periradicular curettage (arrow)

The differential diagnosis included a nasopalatine duct cyst, a periapical cyst, a central giant cell granuloma, and a primordial cyst. The cystic lesion was surgically removed under local anesthesia via a palatal approach. A full-thickness envelope flap was raised, extending from the upper left first premolar to the upper right first premolar. The cystic capsule, adhered to the palatal periosteum, was dissected and the lesion was exposed, while the translucent fluid contained in the cyst discharged spontaneously. The cyst was enucleated and completely removed. The bone cavity was thoroughly curetted with sharp Lucas curettes. Subsequently, the periapical pathology of #22 was intra-operatively visualized and accessed. Using a surgical burr under copious irrigation with sterile saline, 3 mm of root end was resected to facilitate periradicular curettage and complete removal of all soft tissues in the peri-apical area. No root-end preparation nor retrograde filling was performed, as the recent primary orthograde root canal filling seemed sound and no root cracks were visible (Figure 2).­

**Figure 3:**
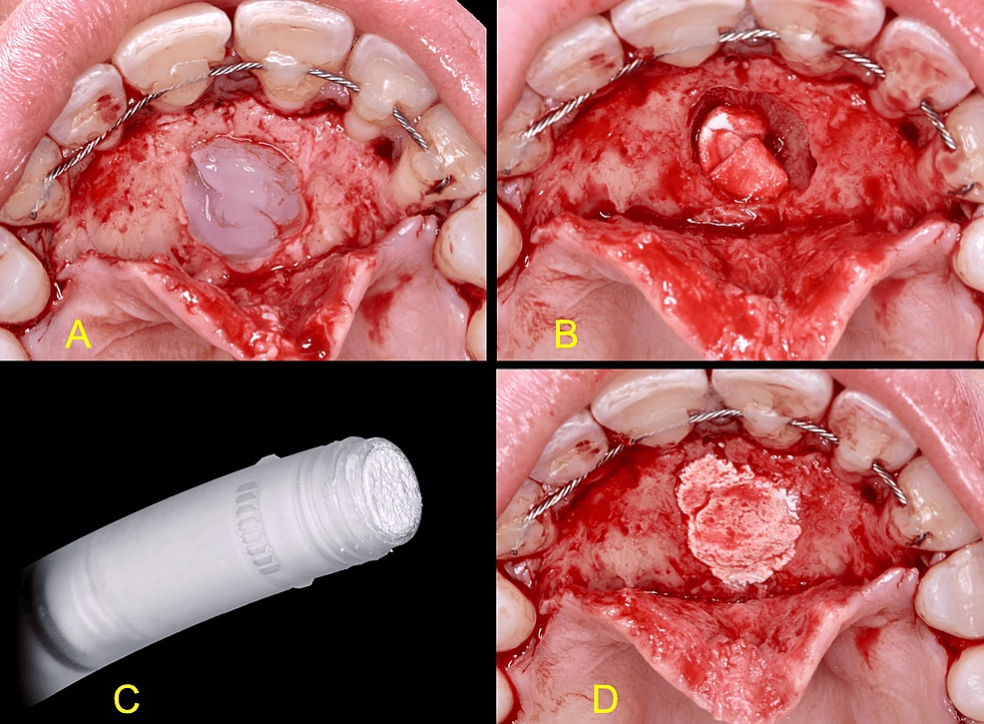
Decontamination and grafting of the bone defect A: Oxygen and lactoferrin-releasing blue®m gel B: Hemostatic collagen placed at the entrance of the incisive canal C: The silicate-substituted β-TCP bone graft (Powerbone Dental Putty). The bone graft is preloaded in a sterile syringe and ready to use. No mixing with sterile saline is required D: The surgical site immediately after bone grafting

The bone cavity was treated for five minutes with oxygen and lactoferrin-releasing oral gel (blue®m, Wapenveld, Netherlands) and then rinsed with sterile saline. Hemostasis was achieved by placing a hemostatic collagen sponge (Hemocollagene, Septodont) at the entrance of the incisive canal, and the bone defect was grafted utilizing a silicate-substituted beta-tricalcium phosphate (β-TCP) fully resorbable alloplastic bone substitute (Powerbone Dental Putty, Bonegraft® Biomaterials). No barrier membranes were used (Figure 3).

**Figure 4:**
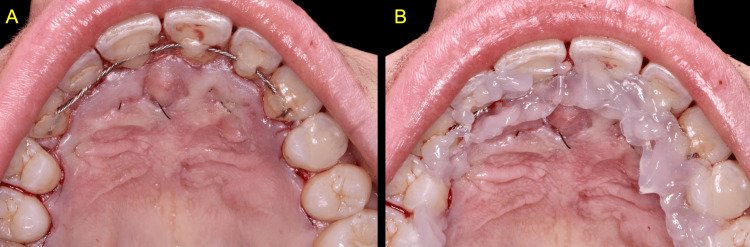
Clinical views immediately post-surgery A: Tension-free primary closure B: Topical application of oxygen and lactoferrin-releasing blue®m oral gel

The mucoperiosteal flap was repositioned and sutured without tension using 5-0 monofilament sutures (SKD® MONO, Miromed), obtaining primary closure. Immediately post-surgery, blue®m oral gel was applied on the sutured surgical wound (Figure 4).

**Figure 5:**
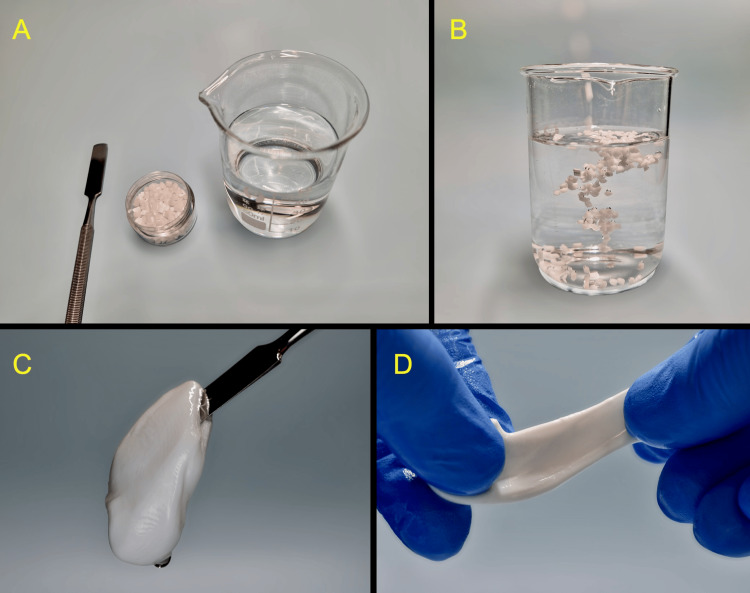
Chairside preparation of the Elemental palatal stent, composed of the zinc-containing thermoplastic surgical polymer A, B: The Elemental granules were placed in hot sterile saline C, D: A soft pliable polymer was formed

A palatal stent, utilizing a novel zinc-containing polymer (Oral Surgical Granulate®, Elemental, Belgium), was fabricated chairside and used for palatal wound protection immediately after surgery and throughout the initial seven-day healing phase. Elemental is based on a patented technology that infuses zinc ions (Zn2+) into surgical thermoplastic polymers. Following the manufacturer’s guidelines, 3 g of Elemental polymer granules were placed in hot (80°C) sterile saline and mixed to form a soft pliable mass (Figure 5).

**Figure 6:**
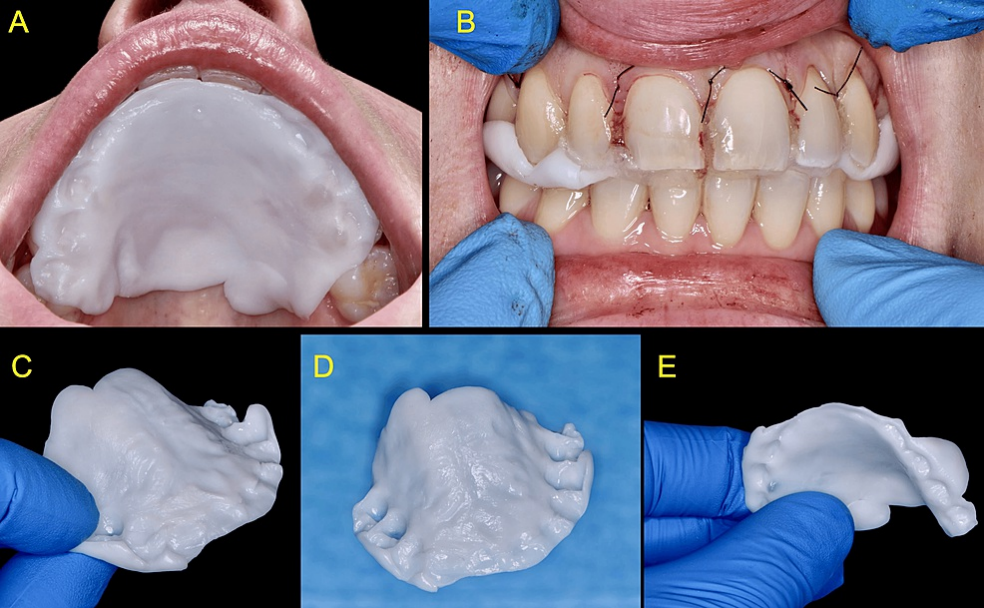
Intra-oral adaptation (A,B) and final shape (C-E) of the zinc-containing Elemental palatal stent. If needed, the stent can be further contoured using surgical scissors

Subsequently, the material was removed from the saline and manually shaped into a uniform layer approximately 2 mm thick. Intra-orally, while still in a moldable state, the polymer was manually applied directly onto the palate and contoured to the palate's anatomy with gentle tactile pressure, ensuring adaptation to the interproximal spaces and undercuts of the upper teeth bilaterally. By further folding the soft polymer over the occlusal surfaces of the upper molars and asking the patient to bite down in centric occlusion, the retention of the stent was improved, and a "handle" was created to enable the patient to remove the stent easily when required. The stent hardened spontaneously in situ after three minutes (Figure 6). 

Ibuprofen 400 mg every six hours and antibiotic therapy consisting of 500 mg amoxicillin every eight hours for five days were prescribed. Blue®m oral gel was provided, with instructions to apply it on the inner surface of the Elemental palatal stent three times daily, ensuring adequate contact time with the healing tissues. The patient was instructed on the proper method for fitting and removing the surgical palatal stent and advised to wear it for seven days, aiming to keep it in place for as many hours per day as possible.

**Figure 7:**
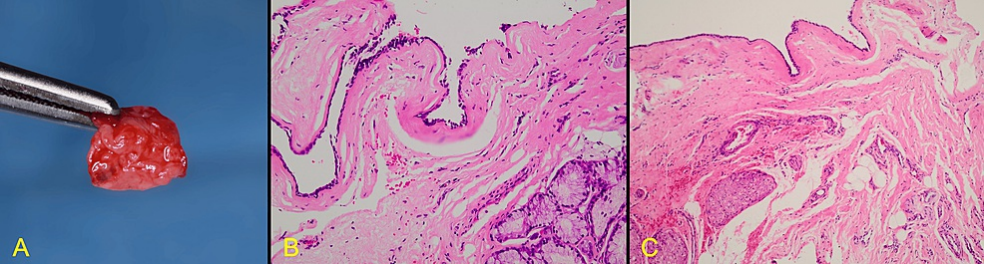
The histopathological evaluation of the lesion established the diagnosis of a nasopalatine duct cyst A: The surgical specimen B, C: Histological pictures

The lesion was fixed in a 10% neutral buffered formalin solution and submitted for histopathological analysis. Histological sections stained with hematoxylin and eosin revealed a cystic lesion with a lining composed of a combination of simple cuboidal and pseudostratified respiratory-type epithelium. The cystic capsule consisted of cellular fibrous connective tissue with few lobules of mucous glands and sections of nerve bundles and vessels. No features of malignancy or inflammation were observed (Figure 7). A histological diagnosis of a nasopalatine duct cyst was established.

**Figure 8:**
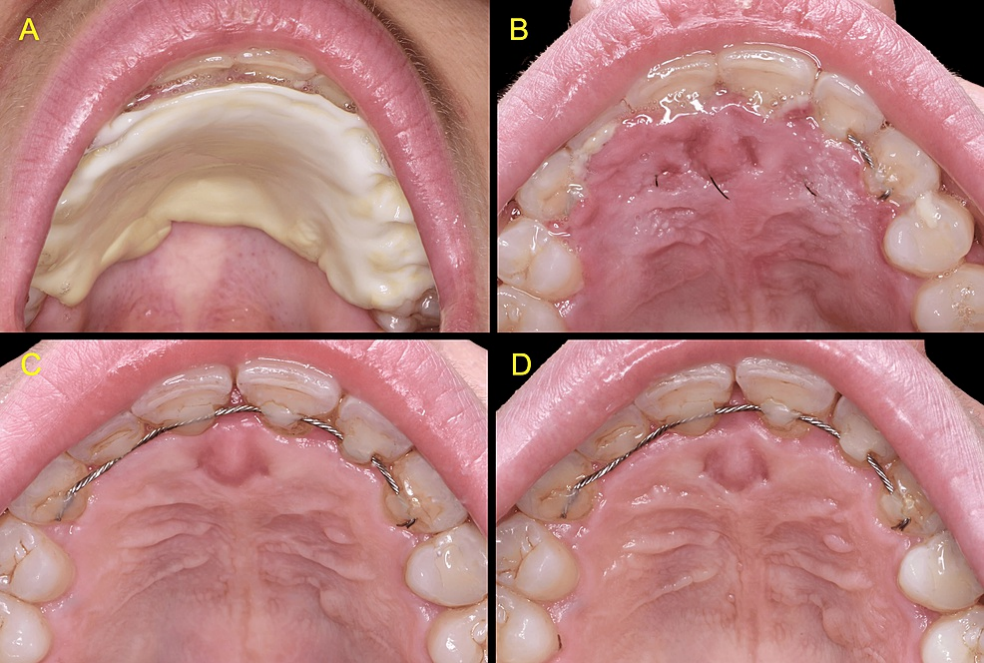
Clinical views at the follow-up appointments A, B: One week post-surgery C: Four weeks post-surgery D: Six months post-surgery

The patient was followed up regularly. Sutures were removed one week after surgery. At this time, the healing was uneventful with minimal soft tissue swelling and no signs of post-operative infection. The patient reported mild pain and used painkillers for the first four days, wearing the zinc-containing palatal stent most of the day during the initial seven days post-operative. The stent facilitated eating and drinking and was easy to remove, clean, and refit with a small amount of blue®m oral gel applied three times daily. The patient discontinued the use of the Elemental stent thereafter. At the four-week follow-up visit, the patient was asymptomatic, and the palatal area exhibited complete soft tissue healing. A follow-up review six months post-operatively revealed excellent healing with no clinical signs of pathology or recurrence of the lesion (Figure 8).

**Figure 9:**
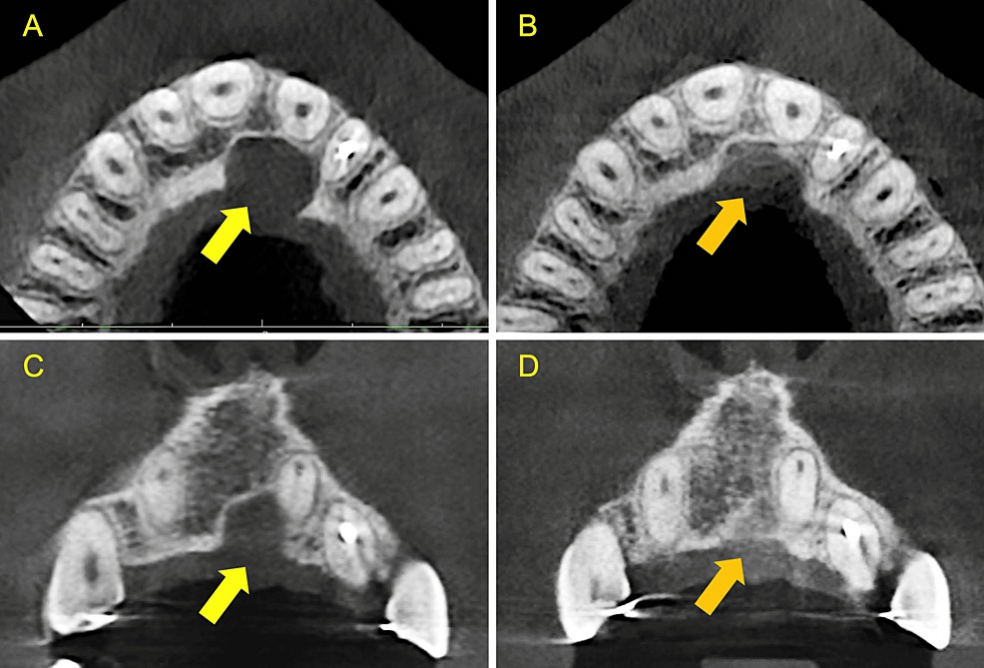
Corresponding radiological views before and six months post-surgery revealing the shrinkage of the cyst A: Axial view pre-operative B: Axial view six months post-surgery C: Coronal view pre-operative D: Coronal view six months post-surgery

There was no hyposensitivity or paresthesia in the anterior palate. The presenting symptoms and signs associated with #22 had been eliminated at this time. Further radiological examination with a CBCT revealed significant shrinkage of the cyst, signs of resorption of the graft material, and simultaneous osseous regeneration (Figure 9).

## Discussion

As illustrated in the present case report, the Elemental palatal stent served as an effective, biocompatible, and comfortable physical barrier that provided excellent mechanical and thermal protection to the palatal surgical wound during the initial seven days after the surgery. As a result, the patient experienced minimal post-operative discomfort, and the follow-up evaluations revealed uneventful healing. In addition, the integration of zinc into the polymer may have contributed to better healing and critical ant-bacterial properties.

In oral surgery, it is crucial to control the biofilms that accumulate and proliferate on the surface of the sutures and the wound margins during the initial healing period in order to minimize the risk of post-operative infections, which can lead to wound dehiscence and impaired healing. Moreover, the adjunctive use of chemical and biological agents that can be incorporated into biomaterials and applied topically, may enhance the healing and facilitate tissue regeneration after surgical trauma, yielding successful and predictable results. The novel formula of the Elemental granulate uses the trace element of zinc in an attempt to fulfill the above purposes. In a randomized controlled clinical trial, Alkaya et al. investigated the effectiveness of chairside-made zinc-containing Elemental palatal stents, compared to suturing of a gelatin-based hemostatic agent, on palatal wound healing and patient morbidity after free gingival graft surgery [9]. The results of this study showed that, in the early phase of palatal wound healing, zinc-containing polymer palatal stents were associated, in a statistically significant manner, with less post-operative pain and bleeding, faster re-epithelialization and shorter surgical times than hemostatic suturing. The favorable clinical results presented in this case conﬁrm the clinical observations made by the above authors.

An oxygen and lactoferrin-releasing oral gel was also utilized as an adjunctive topical therapy to control the biofilm and promote healing. The topical release of oxygen exhibits bactericidal effects and creates an unfavorable environment for the growth of anaerobic bacteria. Additionally, a low, stable, and safe concentration of oxygen delivered at the surgical site can enhance wound healing by stimulating and promoting several essential molecular and cellular processes, including neovascularization, collagen production, re-epithelialization, and degradation of necrotic tissue [10]. Moreover, it has been shown that lactoferrin, a multifunctional glycoprotein, when applied locally may enhance bone regeneration and also support the initial phases of soft tissue healing, providing anti-inflammatory effects that modulate the immune response, improving granulation tissue and collagen formation, and stimulating fibroblast and keratinocyte proliferation and migration [11]. As a result, the local release of oxygen and lactoferrin from the blue®m oral gel, in synergy with the effects provided by the zinc, which is embedded in the stent's polymer, can have a significant impact on biofilm control, wound healing and both soft and hard tissue regeneration, as illustrated in the present case.

The nasopalatine duct cyst, also known as the incisive canal cyst, is the most common non-odontogenic cyst of the gnathic bones, with an incidence ranging from 32.8% to 68.8% [12]. The incisive canal typically has a diameter of up to 6 mm; if the diameter exceeds this measurement, an incisive canal cyst should be suspected [13]. In the present case, the lesion measured 11 mm in diameter. The epidemiological, clinical, radiological, and histopathological features of the nasopalatine duct cyst are widely documented in the literature [12-15], and the features of the lesion observed in this case are consistent with the reported data.

There is no consensus on the etiology of the nasopalatine duct cyst. It is believed that it arises from the proliferation of epithelial remnants of the nasopalatine duct, which is a communication between the nasal cavity and the anterior maxilla formed in the developing fetus. It has also been suggested that the proliferation of these epithelial cell remnants can be induced by irritation, local trauma, or infection [12,13]. In the present case, orthodontic palatal expansion at the age of eight might have been involved in the pathogenesis of the lesion. However, such a hypothesis has not been previously reported in the literature.

Nasopalatine duct cysts are treated with complete surgical removal [13]. Radiological evaluations up to three years post-operatively reveal that a significant percentage of patients (approximately 20-30%) do not heal with complete bone fill. An explanation for this is that during the healing of large nasopalatine duct cysts, fibrotic tissue may develop between the soft tissue of the palate and the floor of the nasal cavity [14,15]. A bone graft was used in the present case to fill the bone defect in an attempt to promote bone regeneration. This decision was also supported by evidence indicating that bone substitutes may enhance bone healing in bone defects after cyst enucleation [16,17]. An alloplastic silicate-substituted β-TCP biomaterial was utilized. Silicate-substituted calcium phosphates possess significant osteoconductive and osteoinductive properties and can promote the regeneration of high-quality vital bone, exhibiting substantial advantages for the repair and regeneration of damaged bone [18]. In the present case, the CBCT at the 6-month follow-up revealed adequate bone regeneration. Taking into account the resorption characteristics of the alloplastic bone substitute used (β-TCP undergoes complete resorption within a period of six to 18 months, depending on individual patient factors and individual osteogenetic bone potential, in addition to factors relating to the materials used such as pore size, porosity, roughness and the incorporation of other chemical elements), it is expected at this time a large portion of the graft to be already dissolved, being gradually replaced by newly-formed provisional matrix, osteoid and bone, holding promise for complete bone fill in the following six to twelve months [19]. It is proposed that a follow-up period of three years is desirable to monitor the healing after the removal of nasopalatine duct cysts [15]. A fully resorbable bone substitute was used in the present case. It is suggested that fully resorbable bone grafts should be used for the management of jaw cysts because filling of bone defects with non-resorbable bone substitutes may lead to alteration of the normal anatomical profile and remodeling failure. Moreover, the use of non-resorbable grafts may also be contraindicated in the case of bony lesions of an unknown entity, as future recurrences might be overlooked or misdiagnosed due to the filler material radiographically mimicking bone regeneration [20].

## Conclusions

The present case illustrates the successful use of a novel zinc-containing palatal stent, in synergy with the beneficial effects of topical oxygen therapy and a fully resorbable synthetic bone substitute, for the surgical management of a nasopalatine duct cyst. The chairside fabrication of this customized stent, as a measure to reduce post-operative discomfort and, in parallel, control the bacteria and enhance the wound healing process, seems to simplify the procedure, reducing both clinical time and costs compared to conventional palatal stents. Additionally, it is of great clinical importance that the integration of zinc nanoparticles into the polymer may provide significant benefits in bacterial control and improvement of early-phase palatal healing following flap surgery in the area. However, further clinical investigations are essential. These should include larger samples and comparison of different materials, to thoroughly validate and supplement the current findings.
